# Targeted NGS: A Cost-Effective Approach to Molecular Diagnosis of PIDs

**DOI:** 10.3389/fimmu.2014.00531

**Published:** 2014-11-03

**Authors:** Jennifer L. Stoddard, Julie E. Niemela, Thomas A. Fleisher, Sergio D. Rosenzweig

**Affiliations:** ^1^Department of Laboratory Medicine, Clinical Center, National Institutes of Health, Bethesda, MD, USA

**Keywords:** primary immunodeficiency, mutation analysis, Sanger sequencing, next-generation sequencing, genotype–phenotype correlation, SNV, INDEL

## Abstract

**Background:** Primary immunodeficiencies (PIDs) are a diverse group of disorders caused by multiple genetic defects. Obtaining a molecular diagnosis for PID patients using a phenotype-based approach is often complex, expensive, and not always successful. Next-generation sequencing (NGS) methods offer an unbiased genotype-based approach, which can facilitate molecular diagnostics.

**Objective:** To develop an efficient NGS method to identify variants in PID-related genes.

**Methods:** We performed HaloPlex custom target enrichment and NGS using the Ion Torrent PGM to screen 173 genes in 11 healthy controls, 13 PID patients previously evaluated with either an identified mutation or SNP, and 120 patients with undiagnosed PIDs. Sensitivity and specificity were determined by comparing NGS and Sanger sequencing results for 33 patients. Run metrics and coverage analyses were done to identify systematic deficiencies.

**Results:** A molecular diagnosis was identified for 18 of 120 patients who previously lacked a genetic diagnosis, including 9 who had atypical presentations and extensive previous genetic and functional studies. Our NGS method detected variants with 98.1% sensitivity and >99.9% specificity. Uniformity was variable (72–89%), and we were not able to reliably sequence 45 regions (45/2455 or 1.8% of total regions) due to low (<20) average read depth or <90% region coverage; thus, we optimized probe hybridization conditions to improve read-depth and coverage for future analyses, and established criteria to help identify true positives.

**Conclusion:** While NGS methods are not as sensitive as Sanger sequencing for individual genes, targeted NGS is a cost-effective, first-line genetic test for the evaluation of patients with PIDs. This approach decreases time to diagnosis, increases diagnostic rate, and provides insight into the genotype–phenotype correlation of PIDs in a cost-effective way.

## Introduction

Primary immunodeficiencies (PIDs) are diseases with variable reported incidence (1/1,200–1/19,000) (1–4), severity, and clinical phenotype. Those at the severe end of the spectrum lead to life-threatening infections and life-limiting complications, thus timely and accurate diagnosis may enable the initiation of specific therapy that can be lifesaving. Unfortunately, obtaining a genetic diagnosis for PID patients is often complex for many reasons including (1) >200 different PID-causing genes have been identified (5); (2) the clinical phenotype within a genotype can vary significantly; and (3) more than one genotype can produce similar clinical phenotypes. One example of the latter is that Mendelian susceptibility to mycobacterial disease (MSMD, MIM **#**209950) can be the result of defects in the genes encoding interferon-gamma receptor-1 (*IFNGR1*), interferon-gamma receptor-2 (*IFNGR2*), the beta-1 chain of the interleukin-12 receptor (*IL12RB1*), interleukin-12 p40 (*IL12B*), signal transducer, and activator of transcription-1 (*STAT1*), as well as other genes.

Today, the most common approach to diagnosing PIDs employs a phenotype-based approach that includes phenotypic and functional characterization followed by Sanger sequencing of one or more candidate genes. This canonical approach can be time consuming, expensive, and may not always lead to a molecular diagnosis. Alternatively, next-generation sequencing (NGS) methods are becoming increasingly accessible in the clinical laboratory setting (6–8). These rapid, accurate, and relatively low cost methods allow a high-throughput, genotype-based approach to molecular diagnosis. For example, targeted sequence enrichment (e.g., Agilent Technologies HaloPlex custom capture kit) followed by NGS sequencing [e.g., Life Technologies Ion Personal Genome Machine (Ion PGM)] (9) provides the ability to rapidly screen large panels of genes. We, therefore, sought to develop a NGS method using HaloPlex target enrichment and the Ion PGM for use as a sensitive and accurate diagnostic tool for simultaneous mutation screening of known or suspected PID-related genes.

## Methods

### Samples

Genomic DNA (gDNA) was extracted from peripheral blood using standard saline extraction methods. Two hundred twenty-five nanograms of gDNA were necessary to perform our test. The validation phases were performed using DNA samples from 11 anonymous healthy adult control subjects (for background filtering) and 13 DNA samples from PID patients with previously identified gDNA mutations or SNPs. The implementation phase involved screening 120 PID patients referred without a genetic diagnosis.

### Capture design

In all, 2455 target regions including the coding exons plus 10 flanking bases of 173 genes known or highly suspected to be associated to particular PIDs (Table S1 in Supplementary Material) (1.23 Mbp) were submitted for DNA capture probe design using the Agilent SureDesign web-based application (https://earray.chem.agilent.com/suredesign/home.htm). Highly conserved intronic regions for three genes (*CTLA4*, *CD28*, and *GATA2*) were also included in the design; however, other non-coding regions, including promoters and other regulatory regions were not included. The final probe design was expected to yield 42,909 amplicons covering 99.53% of the submitted target regions. We were unable to design probes for *IKBKG* (*NEMO*), due to the presence of a pseudogene, or *STXBP2* due to the failure of the SureDesign probe design software to identify probes for >82% of the coding region; these two genes were, therefore, excluded from the evaluation.

### Target enrichment, library preparation, and NGS

Capture of the target regions was performed with reagents from a custom design HaloPlex Target Enrichment kit (Agilent Technologies), according to the HaloPlex Target Enrichment System Protocol. Briefly, the protocol consisted of the following steps: (1) digestion of gDNA with restriction enzymes; (2) hybridization of fragments to probes whose ends are complementary to the target fragments (during this step, fragments are circularized and sequencing and barcode adapters are incorporated); (3) capture of target DNA using streptavidin beads and ligation of circularized fragments; and (4) PCR amplification of captured target libraries.

Quality control of all libraries was performed on the Agilent Bioanalyzer using a High Sensitivity chip. Template dilutions were calculated after library concentrations were normalized to ~100 pM using the Ion Library Equalizer kit (Life Technologies). Library templates were clonally amplified using the Ion One Touch 2^™^, following the manufacturers’ protocol. Recovered template-positive ion sphere particles (ISPs) were subjected to enrichment according to the manufacturer’s protocol. Samples were subjected to the standard Ion PGM 200 Sequencing v2 protocol using Ion 318 v2 chips (Life Technologies). Up to three samples were loaded per Ion 318 v2 chip due to variable coverage uniformity.

### Bioinformatics analysis for NGS results

Mapping and variant calling were performed using the Ion Torrent Suite software v3.6. In short, sequencing reads were mapped against the UCSC hg19 reference genome using the Torrent Mapping Alignment Program (TMAP) *map4* algorithm. The output of sequence alignment is a BAM file containing mapped reads. SNPs and insertions and deletions {INDELS} were called by the Torrent Variant Caller plugin using default germ line, low stringency settings [minimum coverage = 6(SNP)/15(INDEL), minimum coverage each strand 0(SNP)/5(INDEL), minimum variant score = 10, minimum allele frequency = 0.1, strand bias 0.95(SNP)/0.85(INDEL)] to minimize false negatives; however, the use of low stringency settings logically increased the number of known false positives in our datasets. Thus, we identified false positives (i.e., variants that were predicted to be deleterious but were present in more than one healthy control) in the datasets for 11 healthy controls. These known false positive variants [Table S2 in Supplementary Material (BED format)] were then filtered from the patient’s VCF (variant call format) files, using VCFtools v.0.1.11. Only reads that were unambiguously mapped were used for variant calling. Variants were annotated using ANNOVAR (10). Coverage was evaluated using the Torrent Coverage Analysis plugin and the output was further evaluated used in-house, custom Perl scripts.

### Sanger sequencing

Sanger sequencing was performed to confirm 59 variants (including SNPs) detected in 33 patients. gDNA was PCR-amplified using GoTaq polymerase (Promega) and specific primers (primer sequences available upon request). Amplicons were bi-directly sequenced using the Big Dye Terminator version 1.1 cycle sequencing kit and an Applied Biosystems 3130xl Genetic Analyzer (Life Technologies).

### Hybridization optimization

The default HaloPlex probe hybridization protocol includes an initial denaturation step at 94°C for 10 min. We reasoned that adding a 2-min 98°C denaturation would boost read depth and coverage by helping to denature GC-rich and other complex templates. To test this hypothesis, we ran two different samples in parallel using the default and modified (98°C) hybridization protocols. Region-by-region average read depth and percent coverage were compared using linear regression and the Wilcoxon signed-rank test (paired).

## Results

### Coverage

**Figure 1:**
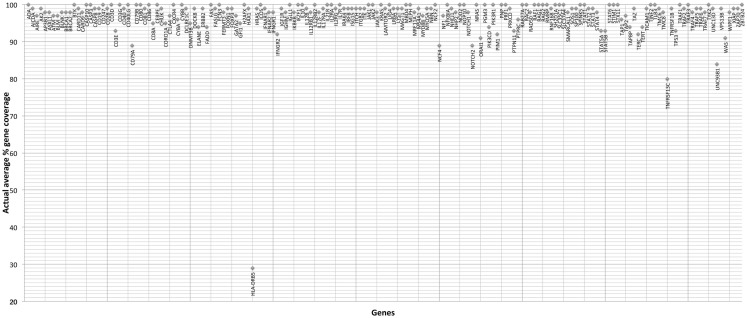
**Gene-by-gene coverage analysis for 33 samples run using the default HaloPlex probe hybridization protocol and ion torrent PGM**.

**Figure 2:**
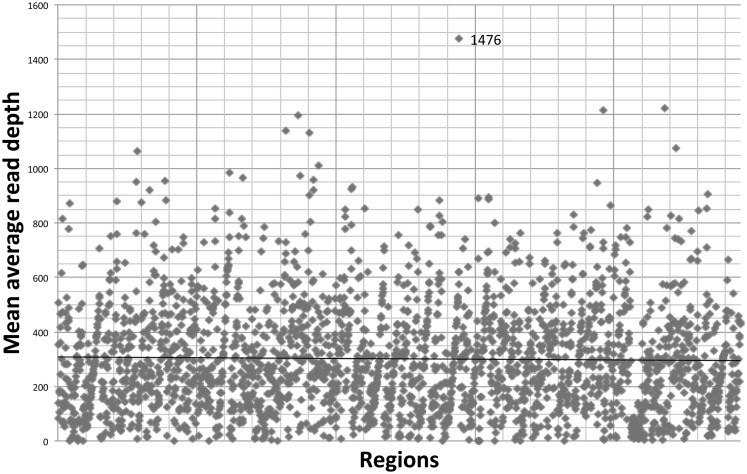
**Region-by region coverage analysis for 33 samples run using the default HaloPlex probe hybridization protocol and ion torrent PGM**.

The gene-by-gene coverage analysis for 33 samples run using the default HaloPlex probe hybridization protocol is shown in Table S3 in Supplementary Material and Figure 1. Although the coverage for *PIK3CD* was expected to be 100%, the actual average coverage was 94% (85–97%). Unfortunately, one of the poorly covered regions contains a known hotspot mutation (*PIK3CD* c.1573G > A, P.E525K). The coverage for following genes was expected to be less than 90% based on the probe design: *HLA-DRB5* (82.95%) and *NOTCH2* (89.43%). Notably, the Haloplex kit is guaranteed to provide >90% coverage for most target regions; however, the actual coverage for following genes was suboptimal (less than 90% and more than 5% lower than expected) (expected/actual): *HLA-DRB5* (83/29), *TNFRSF13C* (100/80), *UNC93B1* (95/84), *CD79A* (100/89), *NCF4* (100/89). The region-by region coverage analysis for 33 samples run using the default HaloPlex probe hybridization protocol (Table S1 in Supplementary Material and Figure 2) demonstrates that multiple regions were systematically poorly covered, including 45 regions (45/2455 or 1.8% of total regions) with low (<20) average read depth and <90% region coverage.

### Run metrics

The run metrics for 30 runs (84 samples) using the default HaloPlex probe hybridization protocol are summarized in Table 1.

**Table 1:** **Run metrics (30 runs, 84 samples)**.

	Chip density%	Total reads per chip	Mean raw accuracy	Q20 bases	Mean read length	Mapped reads	On target%	Mean depth	Uniformity%	Variants
Min	66	3724054	97.8	2106080	72	1147792	64	152	52	74
Max	90	8063266	99.4	398193455	158	3748971	94	921	91	741
Median	77	5859316	99.1	184490059	118	1872132	85	305	85	218
25th percentile	73	5195535	99.0	127030742	113	1553073	80	242	83	150
75th percentile	82	6560932	99.2	236941493	128	2162829	87	425	86	234

### Annotation

All single nucleotide variants (SNVs) were correctly annotated using our in-house bioinformatics pipeline, which utilizes ANNOVAR; however, the notation for INDELS was usually incorrect requiring manual curating. In most cases, it was necessary to perform Sanger sequencing to correctly characterize an INDEL, as characterization of INDELs can be challenging using NGS data.

### Sensitivity and specificity

We established the sensitivity and specificity of our NGS method by comparing the results to those obtained by Sanger sequencing (Table 2 and Table S4 in Supplementary Material). After excluding exon-flanking regions beyond the +5 and −5 positions, which were not reliably detected, the false negative rate was 2% (1 false negative in 52 true positives) indicating a sensitivity of 98.1%. Since intronic variants located >5 bases from splice sites were not reliably detected, we have now remedied this by modifying subsequent capture designs to extend the exon-flanking regions to 25 (from 10) bases. Remarkably, we were able to detect a reversion mutation (*IL2RG* c.460C > T, p.P154S; patient I10) that was present in only 3% of the alleles as the coverage was fairly deep at the variant position (depth = 257); however, this was detected only after reanalyzing the dataset without using an alternate allele frequency cut-off. Nonetheless, our method is not sensitive enough to reliably detect rare somatic alleles due to the non-uniformity of coverage. All of the false negative variants (including the intronic false negative variants) could be attributed to low coverage.

**Table 2:** **Summary of comparison of NGS and Sanger sequencing results (n = 33 samples)**.

True positives	52	True negatives	59012
False negatives	1	False positives	2
False negative rate	1.9	False positive rate	0.003
Sensitivity (exonic)	98.113	Specificity	99.997

The false positive rate was <0.1% (2 false positives among 59,012 true negative bases) indicating a specificity of >99.9%. One of the false positives was also present in datasets from other samples in the same run indicating that it was likely an artifact. The other false positive (*GATA2* c.1-2067C > T) was due to the presence of a homopolymer run. Both false positive changes were ruled out by bidirectional Sanger sequencing.

Based on our experience during the validation and implementation phases of our study, we have identified useful criteria for identifying high probability SNV calls, that is, those calls that are likely to be true positives (Table 3). If a SNV meets these criteria, we feel that Sanger sequencing confirmation is not warranted as the variant is highly likely to be a true positive. Alternatively, all SNVs that do not meet these criteria and all INDELs must be confirmed by an independent method (e.g., Sanger sequencing).

**Table 3:** **Criteria for identifying highly likely true positives**.

Base caller Phred-based *q* score ≥30
Total read depth ≥20
Variant allele frequency ≥25%
Coverage is too low for reliable detection of somatic variants
BAM file should be manually inspected if somatic variant is suspected
Variant is not present in any wild-type controls
Variant is not present in other samples within same run (i.e., samples from patients with different phenotypes)
No other variants (SNVs or INDELS) are present within the same reads containing the variant allele
Variant is not contained within or immediately adjacent to a homopolymer run or repeat region
Variant is not an INDEL
All INDELS must be confirmed by an independent method (e.g., Sanger sequencing)

### Diagnostic efficiency

The diagnostic efficiency of our targeted NGS method for detecting PIDs was demonstrated by our ability to obtain a molecular diagnosis for 18 of 120 (15%) patients who previously lacked a genetic diagnosis, including 9 who had extensive previous genetic and functional studies.

### Hybridization optimization

**Figure 3:**
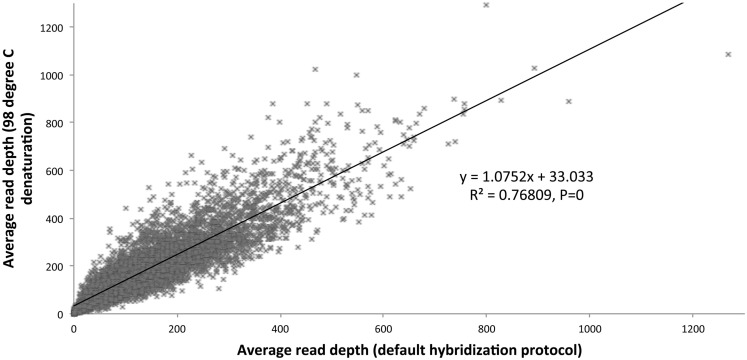
**Average read depth compared (default hybridization protocol vs. addition of 2-min 98°C initial denaturation)**.

**Figure 4:**
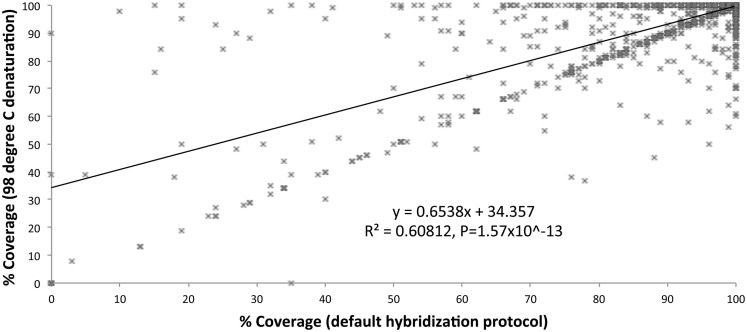
**Percent coverage compared (default hybridization protocol vs. addition of 2-min 98°C initial denaturation)**.

We optimized the HaloPlex probe hybridization by adding of a 2-min 98°C denaturation step. This simple modification boosted median average read depths significantly from 142 to 182 in two samples tested in parallel (Wilcoxon signed-rank test *V* = 18,89,459, *p*-value < 2.2e-16; Table 4; Figure 3). While the median and interquartile range for percent coverage did not appear to be different among protocols, the Wilcoxon signed-rank paired test showed that an increased percent coverage resulted from the optimized protocol (*V* = 52,943.5, *p*-value < 2.2e-16; Table 4; Figure 4). The coverage of some regions was decreased using the modified protocol; however, these regions were fewer than those that showed the same or increased coverage (Figure 4). Notably, the optimized protocol yielded 179 fewer flagged regions (i.e., regions with an average coverage of <20 reads and/or regions that are <100% covered). Unfortunately, the region encoding the PIK3CD E525K hotspot mutation was not rescued by our optimized probe hybridization protocol.

**Table 4:** **Descriptive statistics for average read depth and % coverage per region for two samples run in parallel using the default and optimized hybridization protocol**.

	Default hybridization protocol	Optimized hybridization protocol (2-min 98°C denaturation)	Difference (optimized-default)	Wilcoxon signed-rank test (paired)
Flags^a^	845	666	−179	
Median average read depth	142	182	40	*V* = 1889459, *p*-value <2.2e-16
25th percentile average read depth	75	104	29	
75th percentile average read depth	230	286	56	
Minimum average read depth	0	0	0	
Maximum average read depth	1267	1293	26	
Median % coverage	100	100	0	*V* = 52943.5, *p*-value <2.2e-16
25th percentile % coverage	100	100	0	
75th percentile % coverage	100	100	0	
Minimum % coverage	0	0	0	
Maximum % coverage	100	100	0	
Regions	4784	4784	0	

*^a^Regions with an average coverage of <20 reads and/or regions that are <100% covered were flagged*.

### Targeted NGS vs. Sanger sequencing reagent costs

We compared reagent costs for our targeted NGS method (Haloplex/Ion Torrent PGM) and various Sanger sequencing analyses (Table 5). The reagent cost for our targeted NGS method is approximately $580 per sample, while the reagent cost for Sanger sequencing is approximately $10 per amplicon. Our data show that targeted NGS is a cost-effective alternative to Sanger sequencing in terms of reagent cost for complex diseases requiring >60 amplicons (e.g., DOCK8 deficiency) and for diseases associated with multiple candidate genes [e.g., autoimmune lymphoproliferative syndrome (ALPS), severe combined immunodeficiency (SCID), hyper-IgM syndrom-(HIGM), and MSMD]. It is important to note, however, that this cost analysis does not include the cost of the labor, which far outweighs reagent costs, particularly for low-throughput methods such as Sanger sequencing.

**Table 5:** **Maximal reagent costs of targeted NGS vs. Sanger sequencing of genes included in the sequence capture design**.

Phenotype	Genes (amplicons)	Total amplicons	Sanger sequencing for all listed genes	Targeted NGS (HaloPlex/Ion Torrent PGM)
DOCK8 deficiency	*DOCK8 (62)*	62	$620	$580
Medalian susceptibility to mycobacterial disease (MSMD)	*IFNGR1 (8), IFNRG2 (8), IL12RB1 (17), IL12B (7), ISG15 (3), STAT1 (21), IRF8 (16), GATA2 (15), CYBB (20)*	115	$1,150	
Immunodeficiency with hyper-IgM (HIGM)	*CD40LG (8), CD40 (12), ICOS (5), PIK3CD (18), AICDA (5), NFKBIA (7)*	55	$550	
Autoimmune lymphoproliferative syndrome (ALPS) and ALPS-like diseases	*FAS (9), FASL (4), CASP8 (10), CASP10 (11), NRAS (4), KRAS (5), CTLA4 (8), PIK3CD (18)*	69	$690	
Severe combined immunodeficiency (SCID)	*CD3D (6), CD3E (11), DCLRE1C (29), ORAI1 (6), ADA (12), IL2RG (8), IL7R (14), NHEJ1 (8), PTPRC (40), RAG1 (7), RAG2 (4), JAK3 (18), CORO1A (14),PRKCD (22), AK2 (18)*	199	$1,990	

## Discussion

Our results demonstrate that the Haloplex custom target enrichment in combination with Ion Torrent PGM sequencing provides a sensitive and specific NGS method for the identification of mutations in PID-related genes, overcoming the complexity of the phenotype-based, candidate gene approach. Indeed, this approach will provide more insight into genotype–phenotype correlations for PIDs.

Nonetheless, our study illustrates that identification of disease-causing genetic defects in patients with PIDs using targeted NGS is still a major challenge, requiring protocol optimization, background and quality metrics-filtering, and manual quality control (visual inspection of the alignment file). Although we optimized the HaloPlex probe hybridization protocol, the potential for false negatives due to variable coverage is a cause for concern in the clinical setting. The systematically poor coverage that we observed for some regions was probably due to regional differences in local sequence chemistry, which may include high GC-content, repeat regions, and highly homologous sequences, which confound probe hybridization and read mapping. These are well-recognized limitations of most sequencing methods, including Sanger sequencing; however, in a diagnostic setting, it is imperative to identify which genomic regions have inadequate coverage, especially those exons containing mutational hotspots. All failed regions should be clearly identified in the clinical report, and further evaluation of clinically relevant regions should be performed by Sanger sequencing or another complementary method to exclude pathogenic mutations. Moreover, a targeted NGS approach will only identify defects affecting genes or exon-boundaries regions included in the test panel. Intronic, promoter, and regulatory region associated changes or low copy number somatic variants would not be detected through this method. Likewise, large insertions, deletions, and other chromosomal abnormalities are not detectable by current sequencing strategies including Sanger sequencing and need to be dealt with using techniques that focus on copy number variation. Finally, unless a variant has been previously characterized as associated with a specific PID, functional assays are still necessary to prove causality between a gene variant and a clinical phenotype.

Regarding result confirmation, the American College of Medical Genetics guidelines state that NGS result confirmation is essential when the analytic false positive rate is high or not yet well established, particularly in whole exome and whole genome sequencing approaches (11). For targeted NGS methods, it is practical to analyze multiple normal controls so as to identify platform-specific false positive variants and then filter these variants from subsequent analyses. In our experience, this practice of background filtering resulted in a low FP rate and >99.9% specificity for our targeted NGS method, thereby reducing the need for independent confirmation of SNVs, as long as they the criteria for high probability SNV calls (Tables 2 and 3). All variants that do not meet these criteria, including all INDELS, should be confirmed by an independent method.

Our reagent cost comparison shows that targeted NGS is a cost-effective alternative to Sanger sequencing for complex diseases requiring >60 amplicons (e.g., DOCK8 deficiency) and for diseases associated multiple candidate genes (e.g., ALPS, SCID, HIGM, and MSMD). It is also likely to be efficient for evaluating atypical syndromes that can be associated with mutations in genes typically associated with more classical phenotypes (12). Moreover, it is important to note that the cost of labor needed to perform DNA sequencing and analysis far outweighs reagent costs, and high-throughput NGS methods are associated with reduced labor costs compared to Sanger sequencing. Thus, NGS is a cost-effective alternative to Sanger sequencing for molecular diagnosis of PIDs, except when testing for known family mutations based on a focused evaluation of a single amplicon.

In summary, targeted NGS methods such as the one described above can be used as a cost-effective first-line genetic test for evaluation of new cases of PIDs; however, results should be considered in the context of a region-by-region coverage report, and second line testing to exclude disease-causing mutations should be performed if warranted (i.e., when coverage is poor for a gene that is a good candidate based on phenotype). In many cases, this approach will facilitate diagnosis compared to the phenotype-based approach. The diagnostic yield is likely to be the highest in cases when the clinical presentation is atypical; for patients with PIDs that exhibit a large genotype–phenotype variability or variable penetrance; and for PIDs in which defects in multiple genes can cause the same phenotype.

## Conflict of Interest Statement

The authors declare that the research was conducted in the absence of any commercial or financial relationships that could be construed as a potential conflict of interest.

## Supplementary Material

The Supplementary Material for this article can be found online at http://www.frontiersin.org/Journal/10.3389/fimmu.2014.00531/abstract

## Abbreviations

PID, primary immunodeficiency; NGS, next-generation sequencing; VCF, variant call format; SNV, single nucleotide variant; INDEL, insertion or deletion
